# Sperm Traits Negatively Covary with Size and Asymmetry of a Secondary Sexual Trait in a Freshwater Crayfish

**DOI:** 10.1371/journal.pone.0043771

**Published:** 2012-08-20

**Authors:** Paolo Galeotti, Guido Bernini, Lisa Locatello, Roberto Sacchi, Mauro Fasola, Diego Rubolini

**Affiliations:** 1 Dipartimento di Scienze della Terra e dell'Ambiente, Università degli Studi di Pavia, Pavia, Italy; 2 Dipartimento di Biologia, Università degli Studi di Padova, Padova, Italy; 3 Dipartimento di Bioscienze, Università degli Studi di Milano, Milano, Italy; California State University Fullerton, United States of America

## Abstract

In species where females mate promiscuously, the reproductive success of males depends both on their ability to acquire mates (pre-copulatory sexual selection) and ability of their ejaculates to outcompete those of other males (post-copulatory sexual selection). Sperm competition theory predicts a negative relationship between investment in body traits favouring mate acquisition (secondary sexual characters, SSCs) and investment in ejaculate size or quality, due to the inherent costs of sperm production. In contrast, the phenotype-linked fertility hypothesis posits that male fertilizing efficiency is reliably reflected by the phenotypic expression of male SSCs, allowing females to obtain direct benefits by selecting more ornamented males as copulation partners. In this study, we investigated the relationships between male SSCs and size and quality (viability and longevity) of ejaculates allocated to females in mating trials of the freshwater crayfish *Austropotamobius italicus*. We showed that the relative size of male weapons, the chelae, was negatively related to ejaculate size, and that chelae asymmetry, resulting from regeneration of lost chelipeds, negatively covaried with sperm longevity. Moreover, males allocated more viable sperm to mates from their own rather than different stream of origin. Our findings thus suggest that, according to sperm competition theory, pre-copulatory sexual selection for large weapons used in male fighting may counteract post-copulatory sperm competition in this crayfish species, and that investment in cheliped regeneration may impair ejaculate quality.

## Introduction

When females are polyandrous, both pre- and post-copulatory sexual competition can occur [1], [2], and paternity success of males may be affected by secondary sexual characters (arms or ornaments, SSCs hereafter) as well as by traits influencing fertilizations success. Males can thus win the paternity race by both being more attractive (sexy or strong) and/or more fertile. Sperm competition theory predicts that pre- and post-copulatory forms of sexual selection counteract each other [1], [3]. Specifically, since males should increase their investment in sperm production as sperm competition increases, and the amount of energy needed to produce both SSCs and sperm cells is limited, there should be a negative relationship between investment in ejaculate size/quality and investment in all other reproductive traits, including those that influence mate attraction [1]. In practice, males may face a trade-off between sperm production/fertilization efficiency and mate acquisition [4].

Some empirical studies on insects, fish and birds supported the hypothesis of a negative relationship between exaggerated arms or ornaments and ejaculate size/quality (e.g. [4]–[7]). In contrast, other studies failed to find any sort of association between SSCs and ejaculate traits (e.g. [8]–[11]), and many others revealed instead positive relationships between investment in ejaculate size/quality and investment in secondary sexual characters (e.g. [12]–[18]). In the latter case, according to the phenotype-linked fertility hypothesis [19], male exaggerated SSCs may have evolved as reliable signals of male fertilization efficiency *via* condition-dependency of both ornaments and sperm. Under this scenario, more ornamented/armed males are advertising their ability to provide enough and efficient sperm to fertilize an entire set of ova, and females may directly benefit by choosing such males if they thereby improve the chances of mating with more fertile partners. However, experimental evidence of fecundity benefits associated with female mating preferences for showy males is presently limited or ambiguous [16], [20].

Astacid freshwater crayfish offer a valuable opportunity to study the relationships between weapons (chelae), that provide benefits in intermale conflicts [21], and ejaculate traits, as shaped by sperm competition and/or female cryptic choice. Crayfish have a promiscuous breeding system, which includes multiple mating, sexual coercion, absence of mate guarding and paternal care, and a peculiar mode of external fertilization and sperm competition (see ‘Study organism’ below). In this study, we used the freshwater crayfish *Austropotamobius italicus* (Faxon 1914; Crustacea: Astacidae) as a model to investigate the relationships between male SSCs and size and quality (viability and longevity) of ejaculates allocated to females during mating.

Males and females at maturity (from the third year onward) possess a similar carapace, but male chelae are 25% larger than those of females, because male (but not female) chelae size increases allometrically with carapace length throughout life [21]–[23]. Chelae are used to threaten and attack opponents during inter-male conflicts, as well as to seize, overturn and position females prior to and during copulation [24]; mating is in fact rough and often coercive, since females may resist male advances [25], [26]. Chelae autotomy, an antipredator adaptation enhancing survival chances of individuals [27], commonly occurs in this and other decapods, but regeneration of lost chelipeds leads to a large asymmetry in chelae size, which disfavours males during contests over resources [21], [28], as well as during mating and sperm removal [26]. Thus, male chelae size and symmetry may be the targets of both pre- and post-copulatory sexual selection [21], [26], [29]. In our previous mating experiments that excluded potential confounding effects of male-male competition we did not find any evidence of pre-copulatory mate choice by females, since latency to mating and insemination did not vary according to body size, chelae size or asymmetry [25], [30]. However, a post-copulatory female cryptic choice in the form of differential allocation may occur, since females lay larger eggs but smaller clutches when paired with relatively small-sized, large-clawed males, and larger clutches of smaller eggs for relatively large-sized, small-clawed males [31].

A previous study on a different crayfish population showed that ejaculate size of *A. italicus* increased with female body size and copulation date, but was not correlated with male body size, chelae size or chelae asymmetry when excluding the effect of a few, very large and possibly senescent, individuals [30], [32]. However, sperm traits other than ejaculate size may contribute to paternity, namely sperm viability, longevity and mobility (see [33] and references therein), and male SSCs might reflect these sperm characteristics rather than sperm number.

In this study, differently from previous ones [25], [30]–[32], we conducted mating trials using male and female crayfish assorted at random concerning body and chelae size, to obtain data on ejaculate size and quality while mimicking more natural mating conditions. If the phenotype-linked fertility hypothesis applies to this crayfish species, we predicted a positive relationship between SSCs (chelae size) and sperm traits (ejaculate size, viability and longevity). Alternatively, in line with sperm competition theory, we may expect a negative association between investment in SSCs and sperm traits, since ejaculate size/quality may be traded against investment in pre-copulatory sexual traits, resulting in lower-quality ejaculates for larger-clawed males. In both cases, since regenerating a lost cheliped may compete energetically with spermatogenesis, we expected a negative association between sperm traits and chelae asymmetry.

## Materials and Methods

### Study Organism, Subjects and Housing Conditions

*Austropotamobius italicus* is a long-lived (maximum lifespan is 10–13 years) crayfish native to Italy [34], [35], which reproduces in October–November [36]. Insemination takes place when males attach some spermatophores to the thoracic sternites of females, mainly on a specialized external receptor, the spermatophoric plate. Spermatophores, consisting of vermicular white filaments of variable length (4–9 mm), are composed by two parts: the central sperm mass and a three-layered spermatophore wall ([37]; PG, unpubl. data]. Following ejaculation and spermatophore attachment to the female thorax, this wall rapidly hardens in water and no sperm is released until egg spawning, which occurs within days to weeks from mating [31], thus leaving ample opportunities for multiple mating by both sexes, and hence for sperm competition. In fact, males mating with already mated females feed on spermatophores previously deposited by other males before releasing their own sperm [28], [38], likely leading to a strongly skewed last-male prevalence in paternity [25]. Fertilization takes place externally, when the mass of eggs, accompanied by a copious amount of glair, is extruded from the female thoracic oviducts and passes over the spermatophores. The glair dissolves their hard membrane and brings spermatozoa, which are aflagellate and immotile in decapods [39], [40], into contact with eggs [41].

Sexually mature (>27 mm of carapace length; CL hereafter) male and female crayfish that had not yet mated in the current year (‘virgin’ individuals hereafter) were collected under license of the local authority (Provincia di Piacenza, authorization N. 0067895, issued on September 22, 2010) from seven different streams tributaries of the river Trebbia (N Apennines, Emilia-Romagna, Italy) during September 2010 (194 individuals, sex-ratio 1∶1) and 2011 (212 individuals, sex-ratio 1∶1) before the breeding season began. Sexes were held separately under a natural light∶dark cycle in 150-l opaque plastic jars. Further details on housing conditions are provided in [25], [26], [30], [31]. Crayfish were uniquely marked with a number on the top of the carapace to allow individual identification. CL of individuals of both sexes and right and left chelae length of males were recorded using a digital calliper (accuracy 0.01 mm); as a measure of chelae length we used the maximum chelae length (chelae length hereafter), and only individuals with both chelipeds were considered for mating trials. The occurrence of regenerated chelipeds was noted, and relative chelae asymmetry was calculated as the percentage of the absolute difference in size between the two chelae on chelae length [25], [30], [31]. In our sample, relative chelae asymmetry ranged between 0% and 67%, being on average 9%. This measure of chelae asymmetry was highly repeatable (*r* = 0.99; see [30]) due to large interindividual variation, and was not related to chelae length (*r* = 0.017, *P*>0.80, *n* = 163). At the end of trials, all crayfish were returned to their natal streams.

### Mating Trials

In both years, we formed focal pairs of crayfish by randomly assigning a virgin, receptive female (identified by the whitish colour of mature glair glands along abdominal sternites) to a virgin male. The mean difference between male and female CL of mating pairs was −0.04 mm ±7.98 s.d. (ranging from −15.8 to 18.3 mm, non-significantly different from 0, *t*_162_ = −0.07, *P* = 0.95; Table 1). We used 161 pairs in mating trials: 57 in 2010 (40 females spawned before trials started and became no longer suitable for tests), and 104 in 2011 (two pairs died before the start of experiments). Forty-three pairs were formed by individuals belonging to the same natal stream.

**Table 1:** Body traits of male and female crayfish used in mating trials in the two years of study.

Body traits	2010 (*n* = 57)			2011 (*n* = 164)		
	mean	s.d.	min; max	Mean	s.d.	min; max
Female CL (mm)	40.15	4.65	29.7; 50.5	39.88	4.57	30.3; 52.2
Female body mass (g)	19.35	6.73	8.5; 37	19.30	6.91	9; 45
Male CL (mm)	40.07	6.92	29.4; 51.9	40.10	6.56	28.1; 52.7
Male body mass (g)	22.03	12.54	7.5; 46	23.22	12.64	7; 65
CL difference male-female (mm)	0.08	8.88	−15.2; 18.3	−0.11	7.49	−15.8; 18.1
Male chelae length (mm)	33.96	11.17	19.2; 55.8	34.00	10.149	18.3; 53.3
Chelae asymmetry (mm)	3.06	5.46	0; 29.2	3.11	5.05	0; 31

The onset of breeding activity was determined by preliminary mating trials; the first mating attempts were observed around October 20 in both years. Mating trials took place in the afternoon between 4–11 November 2010 (the delay was due to technical constraints) and between 24 October–9 November 2011. Ten min before a trial started, pair members were placed separately in a 15-l plastic aquarium, provided with a gravel substratum, air stones and an opaque perforated plastic divider, forming two habituation chambers. The divider was then removed and the animals were allowed to freely interact for 60 min. Four pairs were observed at a time under dim red light and their behaviour was recorded by an observer; if copulation and spermatophore deposition (i.e. insemination) did not occur during a mating trial, pair members were returned to their original jar and re-tested in the following days until a maximum of four trials was reached (the same pair was tested on average 1.5 times). Overall, we performed 242 mating trials, and insemination took place in 113 out of 161 crayfish pairs (40/57 in 2010 and 73/104 in 2011).

After insemination, we took two pictures of the ventral parts of each female with a millimetre reference by its side using a digital camera (Panasonic Lumix DMC FZ28, 10.1 Mp sensor resolution, 3.648×2.736 pixels output images) in a standard photographic set (illumination, camera settings and distance of the subject set constant for all pictures). Spermatophores were then removed from female ventral parts and opened within 10 min of collection to determine sperm viability and longevity (see below).

### Measures of Ejaculate Size and Quality

As a measure of ejaculate size we calculated the area covered by spermatophores on the female ventral parts (sperm area hereafter). Spermatophores are mostly deposited horizontally as a single layer on or around the female spermatophoric plate, with limited overlap. Sperm area represents a reliable index of sperm expenditure, as it is strongly positively correlated with both sperm mass (*r* = 0.83, *P*<0.0001, *n* = 49) and total sperm number (*r* = 0.72, *P*<0.0001, *n* = 42; see [42]).

The total area covered by spermatophores was measured in mm^2^ using the image processing and analysis software ImageJ 1.44 [43]. Within a subsample of individuals, sperm area was highly repeatable both within and between pictures of the same female (*r* = 1.0, *F*_9,10_ = 4969.2, *P*<0.0001, and *r* = 0.99, *F*_9,10_ = 2376, *P*<0.0001, respectively).

**Figure 1:**
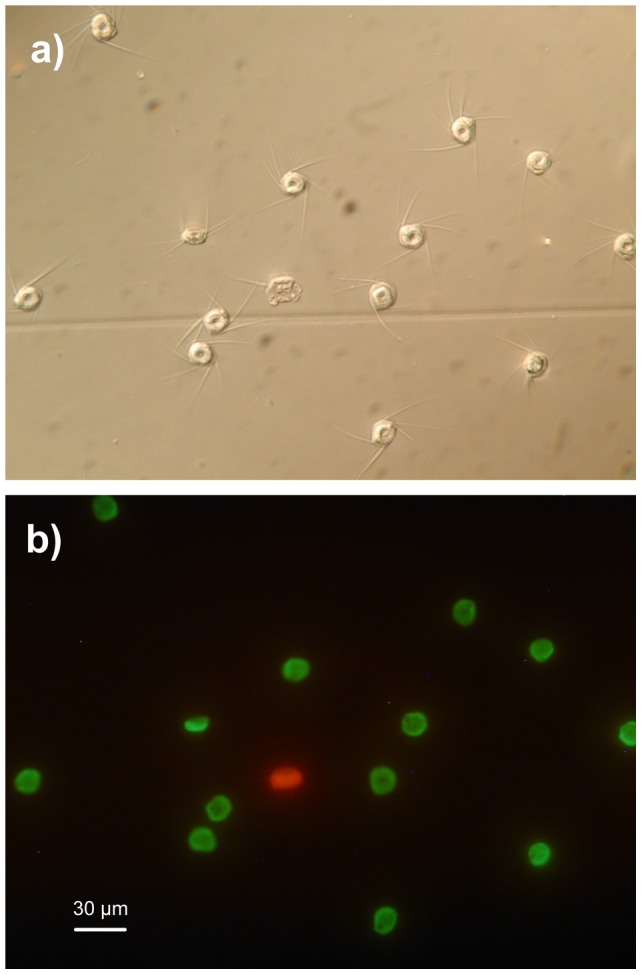
Stained freshwater crayfish sperm cells released from spermatophores (40× magnification). a) Phase contrast microscopy: note the radial nuclear arms or spikes spreading out from the sperm cells at activation; b) Fluorescence microscopy: the same sperm cells under fluorescence light with blue excitation filter (*λ* = 450–480 nm); living sperm with intact cellular membranes are green, while dead sperm are red.

Measures of sperm quality were recorded only on ejaculates collected in 2011, when we developed a reliable method to extract and analyze sperm. Astacid crayfish spermatozoa, which are tightly packed within the spermatophore *lumen*, are large, roundish and aflagellate. At release from opened spermatophores, sperm cells activate by spreading out a variable number of tiny radial arms or spikes (3–9, Fig. 1a), which seemingly serve to passively attach the sperm to the surrounding eggs within the glair emitted by females during spawning [44].

We recorded two measures of sperm quality: sperm viability, expressed as the % of live sperm at extraction from the spermatophore, and sperm longevity, expressed as the % of live sperm at 30 min after extraction from the spermatophore and sperm cell activation. We used the % of live sperm at 30 min as a proxy of sperm longevity because crayfish sperm are non-motile and, thus, sperm longevity cannot be assessed through the traditional measures of duration of sperm motility (e.g. [45], [46]). Two complete, randomly selected, fresh spermatophores from 55 males were placed separately into 100 µl of NaCl 0.9% physiological solution. Using soft tweezers and microlancets, each spermatophore was uncapped under a binocular microscope and gently squeezed to collect two 10 µl sperm samples by a micro-pipette; one sample was immediately analyzed at extraction to assess viability, whereas the other was conserved in an Eppendorf tube at 10°C and processed after 30 min to assess longevity. The latter procedure led to sample decay (sperm agglutination) in 8 cases. Thus, we analyzed two sperm samples at extraction for 55 males and two at 30 min for 47 males.

To assess sperm viability and longevity, we used a live/dead sperm viability assay (Invitrogen Molecular Probes), which stains live sperm green with SYBR-14, a membrane-permeant nucleic acid stain, while dead sperm with damaged membranes is stained red with membrane-impermeant propidium iodide (Fig. 1b). Each sperm sample was added with 3 µl of diluted (1∶25 in DMSO and then 1 10 in distilled water) 1 mM SYBR-14, incubated in the dark for 5 min and mixed with 1 µl of 2.4 mM propidium iodide. The sample was then incubated in the dark for an additional 5 min before being observed in fluorescence microscopy on an “improved Neubauer chamber” haemocytometer. The incubation period for both stains was shortened from 10 to 5 min in order to limit the potential negative effects described for this method [47]. For each sample we assessed the proportion of live sperm over 100 sperm cells counted using an Olympus BX51 microscope equipped with a 100W mercury lamp, at a 40× magnification under a 450–480 nm excitation filter. This measure resulted highly repeatable between the two spermatophores from the same ejaculate both soon after extraction (*r* = 0.98, *F*_9,10_ = 137.4, *P*<0.0001) and after 30 min (*r* = 0.99, *F*_9,10_ = 296.6, *P*<0.0001). Thus, we averaged sperm viability and longevity measured on the two spermatophores, and the mean value was used in the analyses. Descriptive statistics of ejaculate traits are shown in Table 2.

**Table 2:** Ejaculate traits of male crayfish used in this study.

Traits	*n*	mean	s.d.	min; max	CV
Sperm area (mm^2^)	113	33.32	14.93	5.35; 73.7	44.81
Sperm viability (%)	55	67.95	17.43	13.2; 91.6	25.65
Sperm longevity (%)	47	49.31	16.83	8.5; 79.8	34.14

Sperm viability is expressed as the % of live sperm at extraction from the spermatophores, while sperm longevity is the % of live sperm after 30 min of extraction (see Materials and Methods for details). CV =  coefficient of variation.

### Statistical Analyses

All variables expressed as proportions (including relative chelae asymmetry, sperm viability and longevity) were √-arcsine transformed (and thus expressed as degrees) before being included in the analyses. The difference in CL between male and female of mating pairs (CL difference hereafter) was expressed as the absolute value.

Since male chelae length scaled allometrically with CL (type II log-log linear regression of chelae length on male CL: estimate 1.89, 95% c.i. 1.81–1.99) and the two variables were strongly positively related (*r* = 0.95, see also [30]), their simultaneous inclusion in statistical models as independent predictors may result in unreliable parameter estimates. We therefore applied a principal component analysis on log-transformed male CL and chelae length to obtain a measure of chelae size relative to body size (e.g. [48]). The first component (PC1) explained 97.8% of variance and was indeed strongly positively related to both male traits (loading 0.99), thus reliably capturing variance in body size. On the other hand, the second component (PC2), which explained the residual variance, was positively related to chelae length (loading 0.15) and negatively to male CL (loading −0.15); PC2 therefore expressed variation in shape of both chelae and CL: high scores of this component denoted males with relatively large chelae but relatively small CL. We thus considered PC2 as a measure of relative chelae size and included both components as independent predictors in statistical models.

We first evaluated the factors affecting the probability that insemination occurred during a mating trial (probability of insemination hereafter) by using a mixed binary logistic regression including stream of male origin and year as random effects, and stream origin of pair members (same or different stream of origin), number of trials, date of trial (with day 1 = 24 October for both years), PC1 (male body size), PC2 (relative chelae size), female CL, CL difference and male chelae asymmetry as covariates.

To assess the relationships between ejaculate features (sperm area, sperm viability and longevity), female size and male SSCs, we ran mixed model analyses (REML method, degrees of freedom estimated according to the Kenward-Roger method), including stream of male origin and year as random effects, and stream origin of pair members (same or different), date of insemination (with day 1 = 24 October for both years), number of trials, PC1, PC2, female CL, CL difference, and male chelae asymmetry as covariates. In models of sperm viability and longevity, we additionally included sperm area as a possible predictor of these sperm traits. Statistical analyses were performed using SPSS 18.0, R 2.8.1 and SAS 9.1.3 (GLIMMIX procedure) packages. Estimates of regression coefficients are reported together with their associated standard errors.

## Results

Probability of insemination decreased significantly with the number of mating trials a pair effected (−0.80±0.23, *t*_138_ = −3.44, *P* = 0.0008) and marginally non-significantly with increasing CL difference between pair members (−0.092±0.047, *t*_138_ = −1.95, *P* = 0.052). Conversely, probability of insemination was unaffected by date of testing or any other variable (all *P*-values >0.26). These results were strengthened when all non–significant predictors were removed from the model at a time (number of trials: −0.82±0.23, *t*_144_ = −3.53, *P* = 0.0006; CL difference: −0.093±0.046, *t*_144_ = −2.00, *P* = 0.047).

**Figure 2:**
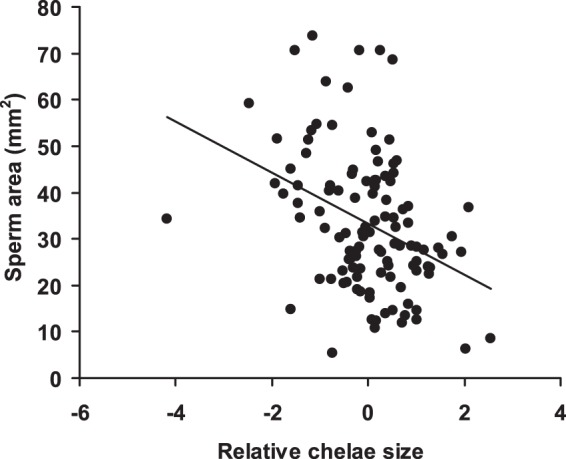
Relationships between sperm area and relative chelae size. Relative chelae size is expressed as PC2 scores (see Methods). The regression line is shown.

**Figure 3:**
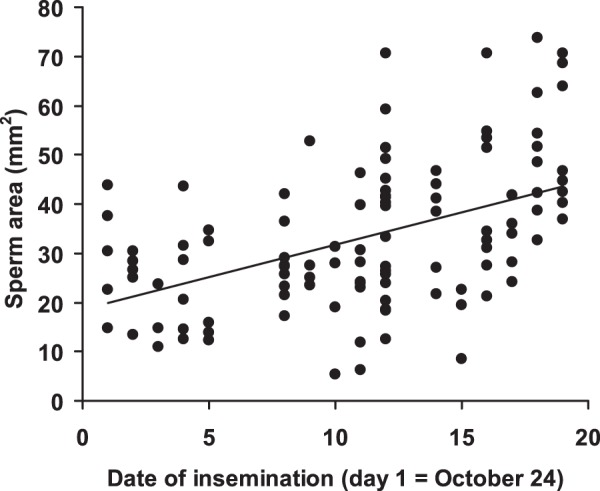
Relationships between sperm area and date of insemination. The regression line is shown.

The mixed model of sperm area showed that PC2 (relative chelae size) was significantly negatively associated with ejaculate size (estimate: −3.99±1.16, *F*_1,102_ = 11.9, *P* = 0.0008). Thus, males with relatively smaller chelae allocated more sperm to females than males with relatively larger chelae (Fig. 2). Sperm area significantly increased with insemination date (*F*_1,23_ = 8.48, *P* = 0.008; estimate: 1.08±0.37; Fig. 3), while no significant effects of all other predictors, including PC1 (relative body size), were apparent (all *P*-values >0.08). Results were qualitatively unchanged when all non-significant predictors were removed at a time from the model (details not shown for brevity).

The mixed model of sperm viability showed a positive, though marginally non-significant, effect of sperm area (*F*_1,45_ = 3.74, *P* = 0.059; estimate: 0.25±0.13), and a significant effect of origin of pair members (*F*_1,45_ = 7.86, *P* = 0.007; estimate: 8.60±3.07). No other terms reached statistical significance (all *P*-values >0.18). The effect of sperm area was however possibly masked by the simultaneous inclusion of PC2 (relative chelae size) and date of insemination, both of which covaried with sperm area (see above). Indeed, when all non-significant predictors were removed at a time from the model, the effect of sperm area on sperm viability became highly statistically significant (*F*_1,52_ = 9.12, *P* = 0.004; estimate: 0.34±0.11), and the significant effect of origin of pair members was confirmed (*F*_1,52_ = 7.14, *P* = 0.010; estimate: 7.82±2.93). Thus, larger ejaculates contained more viable sperm and males ejaculated more viable sperm when mating with females coming from the same stream than when pair members came from different streams.

**Figure 4:**
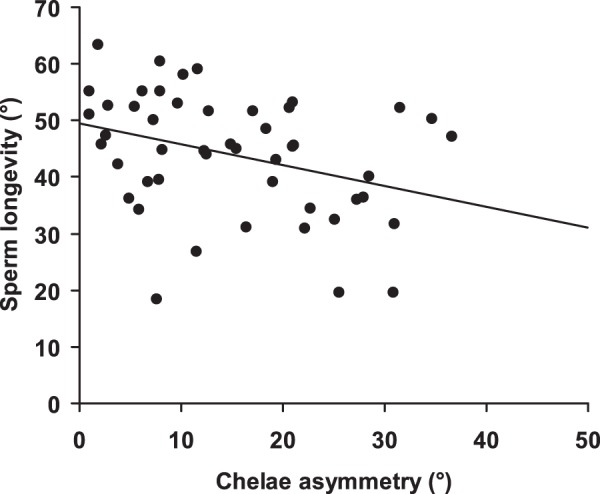
Relationships between sperm longevity and male chelae asymmetry. Both variables were √ arcsine-transformed and are thus expressed in degrees (°) (see Materials and Methods). The regression line is shown.

A mixed model including all variables showed that sperm longevity increased with increasing date of insemination (*F*_1,38_ = 6.42, *P* = 0.016, estimate: 1.04±0.41). Conversely, sperm longevity significantly decreased with increasing chelae asymmetry (*F*_1,38_ = 4.54, *P* = 0.039, estimate: −0.31±0.14; Fig. 4). All other variables did not significantly predict sperm longevity in any model (all *P*-values >0.10). Results were qualitatively unaltered when all non-significant terms were removed from the model at a time (insemination date: *F*_1,45_ = 5.73, *P* = 0.020, estimate: 0.76±0.32; chelae asymmetry: *F*_1,45_ = 6.97, *P* = 0.011, estimate: −0.38±0.14). Thus, males that inseminated females later in the breeding season emitted longer-lived sperm. Conversely, males with highly asymmetric chelae appeared to release shorter-lived sperm.

## Discussion

The main finding of our study was that, consistent with sperm competition theory rather than with the phenotype linked fertility hypothesis (*sensu* [19]), the expression of sexual weapons, chelae size, an androgen-dependent trait in decapods [49], negatively covaried with sperm expenditure, though it did not predict sperm viability or longevity. Therefore, for similarly-sized males, ejaculate size decreased with increasing chelae length. Since ejaculate size was positively related to sperm viability, this suggested that relatively larger-clawed males were also less fertile. In addition, the sperm of highly asymmetric-clawed males, which have been investing energy in regenerating a lost cheliped, showed a reduced longevity compared with those of more symmetric-clawed individuals.

Thus, energy allocation to weapons may competitively reduce energy investment in sperm production and quality, and males with exaggerated SSCs may therefore be incapable of increasing investment in their ejaculates. Alternatively, larger-clawed males could reduce sperm allocation per female in order to fertilize a greater number of partners than smaller-clawed males (e.g. [50], [51]). However, probability of mating was unrelated to male chelae size, and thus larger-clawed males are unlikely to produce smaller ejaculates in order to allocate sperm sparingly among multiple partners. Nevertheless, males growing larger weapons are advantaged in inter-male agonistic encounters as well as in coercive mating [28], [52], but beyond a certain threshold, they may become hindered by these massive structures (chelae in decapods can actually make up 35–50% of their total dry weight; [24]), mainly if males invest more in developing larger chelae, or in regrowing a lost chela, than in producing a high-quality chelae muscle, as for example occurs in male slender crayfish (*Cherax dispar,* [53]). Although favoured in pre-copulatory sexual competition (intimidation or fighting), males with disproportionately large chelae release fewer sperm and thus may be disfavoured by post-copulatory sexual selection either in the form of sperm competition or cryptic female choice. In fact, this handicap may promote cryptic female choice for males that are better able to trade efficiency in pre-copulatory sexual competition (chelae size) with efficiency in post copulatory sexual selection (ejaculate size and quality): males that do not grow chelae disproportionately larger than their carapace could be favoured by post-copulatory sexual selection.

These findings may be reconciled with our previous study documenting female differential allocation to egg and clutch size depending on male traits [31], with females laying larger clutches for relatively large-sized and small-clawed males, possibly maximising their reproductive success since this allocation pattern may lead to an increased production of high-quality offspring (classical differential allocation). On the other hand, females laying fewer larger eggs for small, large-clawed males may be optimizing clutch size to the limited sperm supplies provided by relatively small-sized, large-clawed males. Whether a larger investment in egg size than number reflects a form of maternal favouritism or ‘compensatory’ maternal investment (*sensu* [54]–[56]) remains to be elucidated by determining the fitness payoffs of relatively large-clawed males under natural, competitive, mating conditions.

Another remarkable finding of this study is that male crayfish provided more viable sperm when mated to females of their own natal stream. This surprising allocation strategy may result in favouring genetically more similar females, which may possess co-adapted gene complexes associated with beneficial adaptations to local environmental conditions, thus preventing outbreeding depression [57], [58]. Thanks to a vast array of sense organs (eyes, chemoreceptors, sensory hairs), crayfish males might recognize females of their own stream not only by means of distance (sex pheromone) and tactile chemoreception [44], [59], but also visually, perhaps through variation in body colour linked to stream substrate (PG, pers. obs.; [60]). Recent studies of humans, feral fowl and crickets suggest that males are capable of making rapid adjustments to the quality of sperm they ejaculate [61]–[63]. Male crayfish may adjust sperm viability by means of seminal fluids secreted by *vasa deferentia* during sperm transit, which may function in nourishing and conserving sperm during and after ejaculation, thereby influencing viability of sperm contained in the spermatophores. Alternatively, male crayfish can have sperm in their *vasa deferentia* that vary in age, as for example occurs in the cricket *Acheta domesticus* [64]; in this case, males could be able to allocate their more valuable young viable sperm to copulations that are likely to provide the greater reproductive payoff, and this may occur when mating with females of their own population [58]. Of course, sperm viability could also be affected by the female’s reproductive tract [65], but in *A. italicus* spermatophores are attached externally to female body and sperm contained in spermatophores does not come in contact with the female genital tract (see Introduction). Moreover, in our experimental design, spermatophores remained attached to female sternites for just few minutes after ejaculation, i.e. before female egg release, and it is thus highly unlikely that female body fluids mediated the differences in sperm viability we observed between pairs from the same stream and pairs from different streams.

Probability of insemination did not increase with date of trial, i.e. male and female receptivity was similar all over the breeding season, but sperm investment increased with insemination date. The increase in sperm expenditure with breeding season we observed in this study is not surprising, since in our previous experiments we found that both ejaculate size and duration of copulation increased with date of insemination [30]. Interestingly, also sperm longevity increased with insemination date. Longevity of sperm after release from spermatohores may be the key component of fertilization success in a species where sperm are immotile and may take hours to reach eggs. At the end of breeding season, most females are ready to spawn and the lag between insemination and spawning may be very short (even a few minutes after insemination), thereby reducing female chances of further mating, as males rarely approach and attempt to mate with spawning females [28], [38] and lowering the risk for last-mating males of sperm removal by rivals. Thus, for males copulating with virgin females the risk of sexual interference (total removal of spermatophores, see [25]) or sperm competition (partial removal of spermatophores) should be higher at the start of breeding season, when weeks may pass between insemination and egg spawning, and females may repeatedly mate with other males in the meantime [25], [31]. Moreover, spermatophores attached to female ventral side may also be consumed by the attrition with substrate during locomotion. Under this scenario, males are expected to forgo copulation or invest fewer and lower-quality sperm in risky matings early in the breeding season, when the opportunity of future mating is higher. Conversely, the risk to have their own sperm removed or lost should be lower for males copulating at the end of the breeding season, when also opportunity for further mating substantially declines [66]. Hence, a male strategy to increase own chances of paternity should be to allocate the best-quality ejaculates late in the breeding season to females which are in pre-spawning condition, as this study showed. Admittedly, this strategy may be mainly or only played on by large, dominant males that can easily monopolize larger females and for a longer time compared to smaller males. This may happen because large males may actively displace the latter from copulation or limit their access to females in competitive contexts, thus potentially increasing their own mating opportunities [28], [30], [52]. This pattern of sperm allocation deviates from theoretical models of sperm competition, which predict increased sperm expenditure under sperm competition risk [1], but is consistent with the strongly loaded raffle occurring among males (and sperm) of this crayfish species. Actually, even a single rival is able to completely remove or significantly reduce previously deposited spermatophores [25], and males do not gain any advantage by increasing their sperm expenditure with increasing risk of sperm competition, because the more sperm they release, the more is removed by subsequent mating males [26].

Finally, the probability of insemination was higher on the first attempt, declining over successive trials, and varied according to body size difference between partners. The relative size of partners may be important in shaping mating decisions in this species. Males may have greater difficulties in seizing and assuming the correct copulation position when mating with females much smaller than them, while, on the other hand, a male much smaller than his partner hardly or never achieves any mating if the female resists (PG, pers. obs.). Taken together, these findings confirm that males and females do not mate indiscriminately [28], [52], but if the pair is not well assorted by size, mating may not occur at all or be delayed until the end of the breeding season.

In conclusion, this study highlighted some aspects of the sexual behaviour of *A. italicus* that may contribute to our understanding of selective forces shaping mating strategies, including the role of male SSCs in post-copulatory sexual selection and female preference. According to sperm competition theory [1], [67], post-copulatory sexual selection may counteract the effects of pre-copulatory sexual selection in this crayfish species. Inter-male competition for mating and the coercive mode of copulation may have driven, in a seemingly Fisherian runaway fashion, the evolution of exaggerated armaments to the point that some males became handicapped in sexual competence.
